# Hypoxia-targeted triple suicide gene therapy radiosensitizes human colorectal cancer cells

**DOI:** 10.3892/or.2014.3238

**Published:** 2014-06-06

**Authors:** HUNG TSUNG HSIAO, LIGANG XING, XUELONG DENG, XIAORONG SUN, C. CLIFTON LING, GLORIA C. LI

**Affiliations:** 1 Department of Radiation Oncology, Memorial Sloan-Kettering Cancer Center, New York, NY 10065, USA; 2 Department of Medical Physics, Memorial Sloan-Kettering Cancer Center, New York, NY 10065, USA; 3 Department of Radiation Oncology and Shandong Key Laboratory of Radiation Oncology, Jinan, Shandong 250117, P.R. China; 4 PET-CT Center, Shandong Cancer Hospital and Institute, Jinan, Shandong 250117, P.R. China

**Keywords:** hypoxia, herpes simplex virus-1 thymidine kinase, cytosine deaminase, uracil phosphoribosyltransferase, colon cancer, radiation

## Abstract

The hypoxic microenvironment, an important feature of human solid tumors but absent in normal tissue, may provide an opportunity for cancer-specific gene therapy. The purpose of the present study was to investigate whether hypoxia-driven triple suicide gene TK/CD/UPRT expression enhances cytotoxicity to ganciclovir (GCV) and 5-fluorocytosine (5-FC), and sensitizes human colorectal cancer to radiation *in vitro* and *in vivo*. Stable transfectant of human colorectal HCT8 cells was established which expressed hypoxia-inducible vectors (HRE-TK/eGFP and HRE-CD/UPRT/mDsRed). Hypoxia-induced expression/function of TK, CD and UPRT was verified by western blot analysis, flow cytometry, fluorescent microscopy and cytotoxicity assay of GCV and 5-FC. Significant radiosensitization effects were detected after 5-FC and GCV treatments under hypoxic conditions. In the tumor xenografts, the distribution of TK/eGFP and CD/UPRT/mDsRed expression visualized with fluorescence microscopy was co-localized with the hypoxia marker pimonidazole positive staining cells. Furthermore, administration of 5-FC and GCV in mice in combination with local irradiation resulted in tumor regression, as compared with prodrug or radiation treatments alone. Our data suggest that the hypoxia-inducible TK/GCV+CDUPRT/5-FC triple suicide gene therapy may have the ability to specifically target hypoxic cancer cells and significantly improve the tumor control in combination with radiotherapy.

## Introduction

Hypoxia is a major determinant of the malignant progression of tumor cells and their response to therapy (1). Clinical studies have shown a strong correlation between tumor pO_2_, local control and overall survival (2,3). Since the vast majority of human solid tumors have median pO_2_ levels lower than that of the surrounding normal tissues (4), efforts have been made to exploit it as a target for cancer treatments. At the molecular level, hypoxia is a powerful modulator of gene expression. An important mediator of hypoxic response is the interaction of a transcriptional complex termed hypoxia inducible factor (HIF) with its cognate DNA recognition site, the hypoxia-response element (HRE) (5,6). The HRE sequences isolated from oxygen-responsive genes have been shown to selectively induce gene expression in response to hypoxia when placed upstream of a promoter, and hypoxia-targeted gene therapy can be achieved (7,8).

Two well-established gene-directed enzyme prodrug therapy strategies, in which Herpes simplex virus 1-thymidine kinase (HSV1-TK) or cytosine deaminase (CD) was used in combination with prodrug ganciclovir (GCV) or 5-fluorocytosine (5-FC) respectively, have been proposed for hypoxia-driven gene therapy in different tumor models (9,10). Double suicide gene combined with HSV1-TK/GCV and CD/5-FC resulted in enhanced anticancer effects and has been used in clinical trials (11,12). However, hypoxia-targeted double suicide gene therapy has not been reported. Studies from us and other groups have demonstrated that co-transduction of uracil phosphoribosyltransferase (UPRT) with CD gene to cancer cells improved antitumor efficacy (13,14). Furthermore, our studies demonstrated that hypoxia-targeted expression of bifunctional suicide gene CDUPRT enhanced the cytotoxicity of 5-FC treatments and radiosensitization under hypoxic conditions (15). We also found the superior antitumor effects of triple suicide gene approach-TKCDUPRT over double gene approach-TKCD in a rat prostate tumor model (16).

In the present study, we generated a human colorectal cancer model expressing triple suicide gene (HSV1-TK, CD and UPRT) under the control of the hypoxia inducible promoter. Significant radiosensitization effects were demonstrated both *in vitro* and *in vivo* with GCV and 5-FC treatments. Our data suggest that the hypoxia-inducible triple suicide gene therapy has the ability to specifically target hypoxic cancer cells and significantly improves the tumor control in combination with radiotherapy. This novel model also appears to be a valuable tool to study tumor hypoxia, radiation effects and hypoxia-targeted radio-gene therapy.

## Materials and methods

### Cell culture

Human colorectal cancer HCT-8 cells obtained from the American Type Culture Collection (Manassas, VA, USA) were grown in RPMI-1640 medium (Mediatech, Herndon, VA, USA) supplemented with 10% fetal bovine serum (FBS; Gemini Bio Products, West Sacramento, CA, USA), 100 U/ml penicillin and 100 μg/ml streptomycin (Gemini). For normoxic culture, cells were incubated in a humidified atmosphere containing 21% O_2_ and 5% CO_2_; for hypoxic culture, cells were incubated in an Invivo_2_ 400 Hypoxic workstation (Ruskinn Inc., Cincinnati, OH, USA) with a gas mixture of 0.5% O_2_, 5% CO_2_ and 94.5% N_2_.

**Figure 1:**
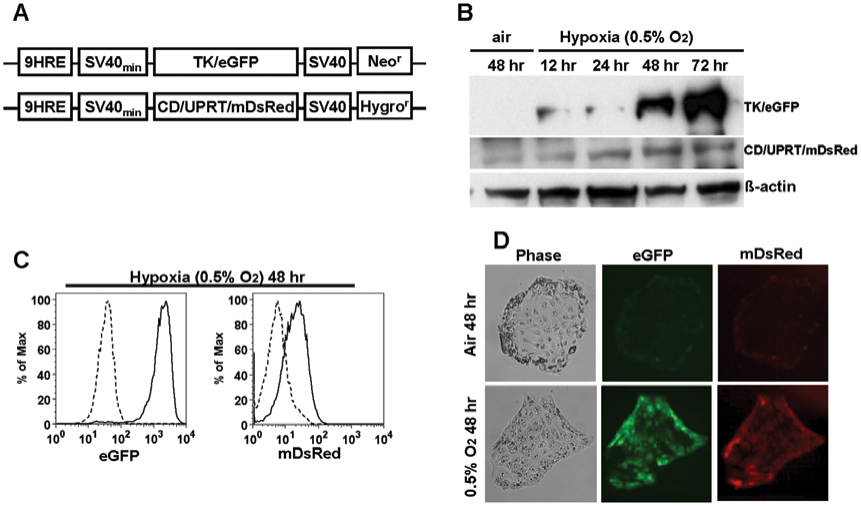
Characterization of HCT8-9HRE-TK/eGFP-CD/UPRT/mDsRed cell lines *in vitro*. (A) Simplified diagrams depicting the 9HRE-TK/eGFP and 9HRE-CD/UPRT/mDsRed constructs. (B) Western blot analysis of TK/eGFP (65 kDa) and CD/UPRT/mDsRed (73 kDa) protein expression under normoxic and hypoxic conditions (0.5% O_2_ for 12–72 h). (C) Flow cytometric analysis of hypoxia-induced eGFP and mDsRed expression. The cells were incubated in air or 0.5% O_2_ for 48 h. The expression of eGFP and mDsRed in hypoxia was displayed in histogram (solid lines) and compared with normoxic cells (dash lines). (D) eGFP and mDsRed expression under fluorescent microscopy in HCT8-HRE cells incubated under either air (upper panel) or 0.5% O_2_ conditions for 48 h (lower panel).

HCT-8 cells were co-transfected with two plasmids; p9HRE-TK/eGFP, which contains the TK/eGFP fusion gene under the control of a hypoxia-inducible promoter and a constitutively expressed neomycin-resistance gene (Neo^r^) (17), and p9HRE-CD/UPRT/mDsRed, which contains the CD, UPRT and mDsRed fusion gene under the regulation of the hypoxia-inducible promoter and a hygromycin B-resistance gene (Hyg^r^) (15) (Fig. 1A). The hypoxia-inducible promoter consists of nine tandem repeats of the HRE from human erythropoietin gene linked to the SV40 minimal promoter (SV40min). The stable cell line expressing HRE-regulated TK/eGFP and CD/UPRT/mDsRed was established by fluorescence-activated cell sorting using the cell sorter (MoFlo; Dako, Carpinteria, CA, USA) (17) and designated as HCT8-HRE cells. HCT-8 cells transfected with empty vector were used as the control cells.

### Western blot analysis

The cells were incubated under normoxic or 0.5% O_2_ conditions for 12, 24, 48 or 72 h, and then whole cell extracts were prepared. The protein was detected with the sheep anti-yCD polyclonal antibody (Biotrend Chemikalien GmbH, Cologne, Germany) and the anti-HSV1-TK monoclonal antibody (kindly provided by Dr W.C. Summers, Yale University, New Haven, CT, USA).

### Flow cytometry

After incubation under normoxic or 0.5% O_2_ conditions for 48 h, the cells were trypsinized, centrifuged and re-suspended in PBS at 4°C. The fluorescence of eGFP and mDsRed was measured using the cell sorter (MoFlo). The data were analyzed with the FlowJo program (Tree Star Inc., Ashland, OR, USA).

### Fluorescence microscopy

The cells were incubated under normoxic or 0.5% O_2_ conditions for 48 h. The cells were then fixed with freshly prepared 4% paraformaldehyde for 10 min and rinsed twice with PBS. The fluorescent images were acquired with a fluorescence microscope (Axiovert 200M, Zeiss).

### Colony formation assay

The cells were treated with 5-FC (InvivoGen, San Diego, CA, USA) or GCV (Sigma-Aldrich, St. Louis, MO, USA) at various concentrations under normoxic or 0.5% O_2_ conditions for 48 h. The cells were then trypsinized, counted, serially diluted and plated into 60-mm dishes. Clonogenic survival was determined by counting crystal violet-stained colonies ~14 days later. The survival fraction was normalized to the cell survival without drug treatments and plotted as a function of the drug concentration.

### MTT assay

The cells were plated into 96-well plates (10^4^/well) and incubated with various concentrations of GCV and/or 5-FC for 48 h under normoxic or 0.5% O_2_ conditions. The medium containing the drug was then replaced with fresh medium, and cells were cultured for an additional 48 h under the normoxic conditions. Tetrazolium dye (Sigma) was added and allowed to react for 2 h at 37°C. After dissolving in DMSO, dye conversion was read using an ELISA plate reader at 490 nm against 610 nm.

### Bystander effect

The HCT-8 control cells were mixed with HCT8-HRE cells at the 80:20 ratio and plated into 96-well plates. The cells were treated with various concentrations of GCV and/or 5-FC for 48 h under 0.5% O_2_ conditions, and cell viability was determined with the MTT assay described above.

### Radiation survival assay

The cells were exposed to 5-FC (0.5 mg/ml) and GCV (5 μg/ml) under normoxic or hypoxic conditions for 48 h, and then irradiated for various doses using a Cs-137 unit (Mark 1 model 68; J L Shepherd & Associates, San Fernando CA, USA) at ~2.0 Gy/min. Clonogenic survival was then determined by colony formation assay. The radiation survival fractions were normalized for plating efficiency using non-irradiated cells with or without drug treatment and plotted as a function of radiation doses.

### Xenograft model

Animal protocols were approved by the Institutional Animal Care and Use Committee. Xenografts were formed by injecting 5×10^6^ cells subcutaneously into the hind legs of 6–8-week old female nude mice (athymic nu/nu; NCI Frederick Cancer Research Institute, Frederick, MD, USA). When the xenografts reached ~10 mm in diameter, the mice were injected i.v. with pimonidazole (80 mg/kg; HPI, MA). Two hours later, Hoechst 33342 (25 mg/kg; Sigma-Aldrich) was i.v. injected, and 1 min later, the mice were sacrificed by CO_2_ breathing. Then, tumors were excised and snap-frozen in OCT mounting medium (Sakura Finetek USA Inc., Torrance, CA, USA). Frozen sections of 8-μm thickness were prepared for the immunohistochemical analysis.

### Immunohistochemical staining and fluorescent microscopic imaging

Tumor sections were first imaged for the eGFP, mDsRed and Hoechst 33342 fluorescence. They were then stained for pimonidazole using FITC-conjugated murine anti-pimonidazole monoclonal antibody (17). Finally, the sections were stained with hematoxylin-eosin with the standard protocol. Fluorescence images were acquired at ×50 magnification using the fluorescence microscope (Axiovert 200 M) equipped with a CCD digital camera, a computer-controlled motorized stage and MetaMorph 7.0 Imaging software (Molecular Devices, Sunnyvale, CA, USA).

### Tumor growth delay

Each tumor was measured with digital caliper in three orthogonal dimensions (a, b and c), and tumor volume was calculated as πabc/6. When the tumor grew to ~100–150 mm^3^, the mice were divided into four groups (10 mice/group) and treated with: i) PBS, ii) 5-FC (i.p. 500 mg/kg) + GCV (i.p. 30 mg/kg) daily for 14 days, iii) radiation (15 Gy) on the 5th day, iv) 5-FC+GCV daily for 14 days followed by radiation (15 Gy) on the 5th day, respectively. The tumor volume was measured three times a week until the volume exceeded 1,000 mm^3^ and plotted as a function of time.

### Statistical analysis

Averages are presented as the mean ± SE. Difference in cytotoxicity and radiosensitivity was determined using the Student’s t-test. A P-value of <0.01 was considered to indicate a statistically significant difference.

## Results

### Hypoxia-induced expression of triple suicide gene

HCT-8 stable cell lines expressing HRE driven TK/eGFP and CD/UPRT/mDsRed were successfully established. Several stable transfectants were established and their gene expressions were similar as verified by western blot analysis, flow cytometry, hence one of them was used for further experiments. Western blot analysis showed that the expression level of TK/eGFP and CD/UPRT/mDsRed protein was markedly increased after hypoxic treatments in a time-dependent manner (Fig. 1B). Exposure of the HRE-TKCDUPRT cells to hypoxia led to an increase of the hypoxia-regulated eGFP and mDsRed fluorescence. In flow cytometric analysis, an 80-fold increase in eGFP and 4-fold increase in mDsRed fluorescence was observed in cells treated at 0.5% O_2_ for 48-h hypoxia compared to normoxic control cells (Fig. 1C). The fluorescent microscopic images of hypoxic and normoxic cell showed that strong eGFP and mDsRed fluorescence signal was observed only in hypoxic cells, confirming the results of flow cytometric analysis (Fig. 1D).

### Hypoxia-induced TK/CD/UPRT expression sensitizes HCT8-HRE cells to 5-FC and GCV

**Figure 2:**
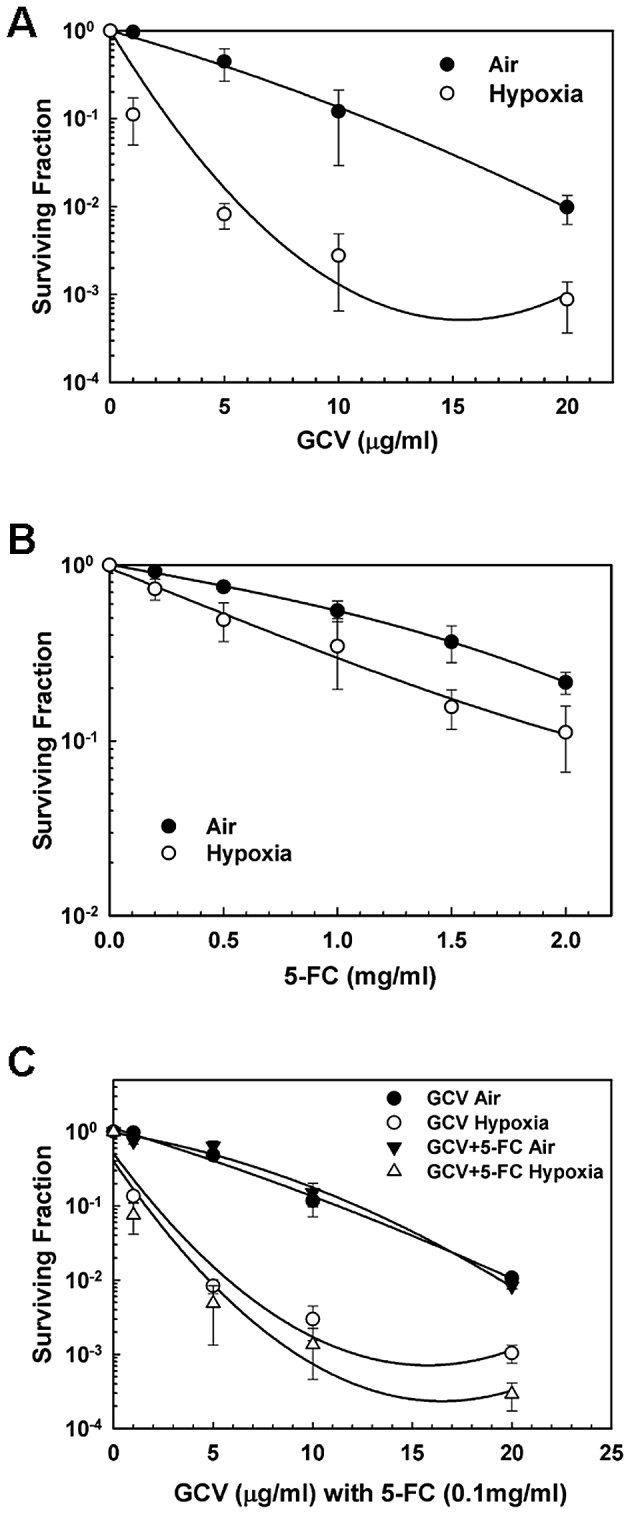
GCV and 5-FC cytotoxicity *in vitro*. HCT8-HRE cells treated at various concentrations of (A) GCV, (B) 5-FC or (C) GCV plus 5-FC under 0.5% O_2_ or normoxic conditions for 48 h and cell survival were determined by colony formation assay. The surviving fractions were normalized to that without drug treatments and plotted as the function of the drug concentration. The error bars are the standard errors from three independent experiments.

**Figure 3:**
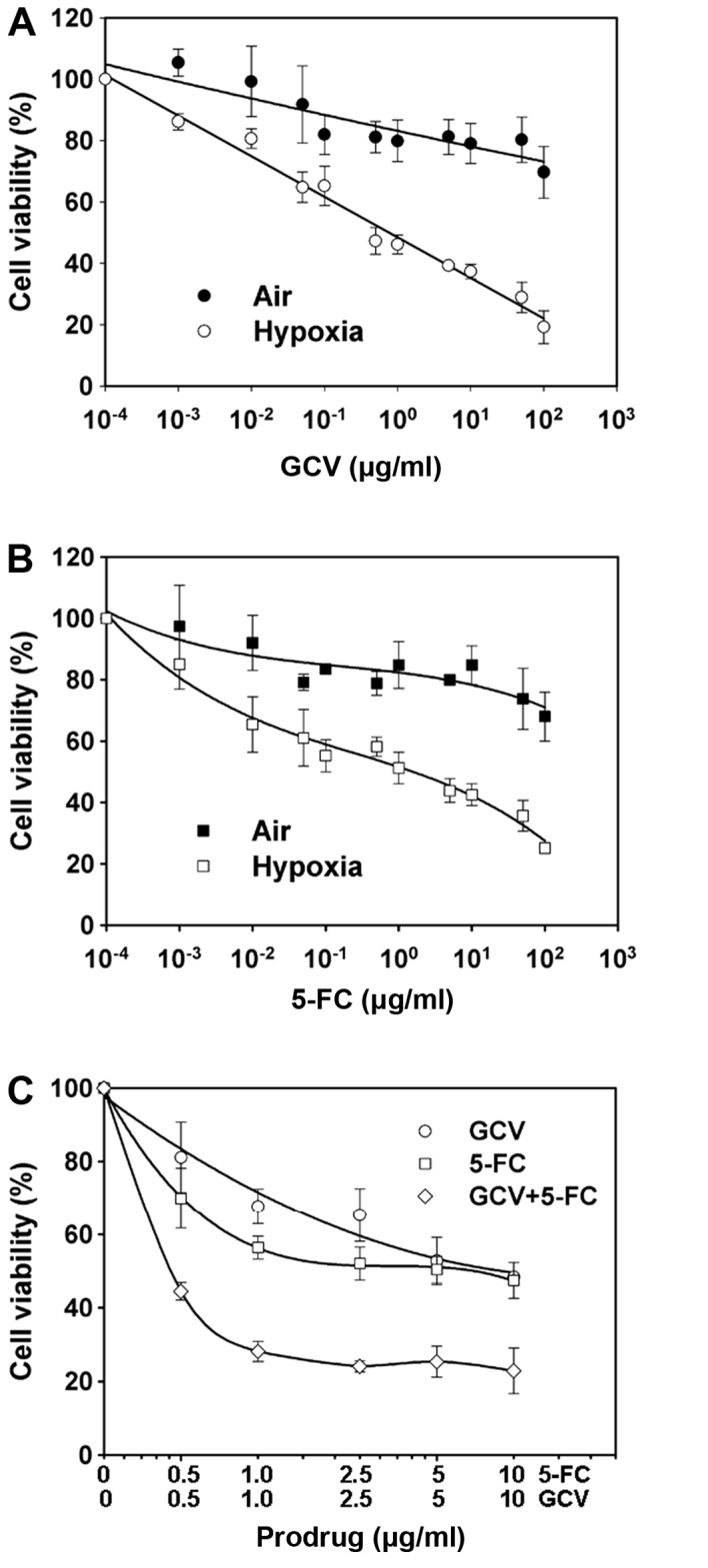
GCV and 5-FC cytotoxicity *in vitro* by MTT assay. The cells were exposed to (A) GCV, (B) 5-FC alone or (C) GCV plus 5-FC under 0.5% O_2_ for 48 h, and cell viability was determined by MTT assay. The cell viability ratios were normalized to that without drug treatments and plotted as the function of the drug concentration. The error bars are the standard errors from three independent experiments.

It was clearly shown that the cell clonogenic survival was significantly inhibited by GCV (Fig. 2A) or 5-FC (Fig. 2B) under hypoxic conditions compared to normoxic conditions. Whereas HCT8-HRE cells were sensitive to GCV and 5-FC administered independently, concurrent prodrug treatment resulted in much greater cytotoxicity (Fig. 2C). Another method, MTT assay, was used to further assess hypoxia-induced cytotoxicity to GCV and 5-FC. It was found that cell viability under hypoxic conditions was significantly inhibited by GCV or 5-FC, relative to that under normoxic conditions (Fig. 3). It was demonstrated again that concurrent prodrug treatment resulted in much greater cytotoxicity.

### Hypoxia-induced expression of TK/CD/UPRT radiosensitizes HCT8-HRE tumor cells pre-treated with prodrug 5-FC and GCV

**Figure 4:**
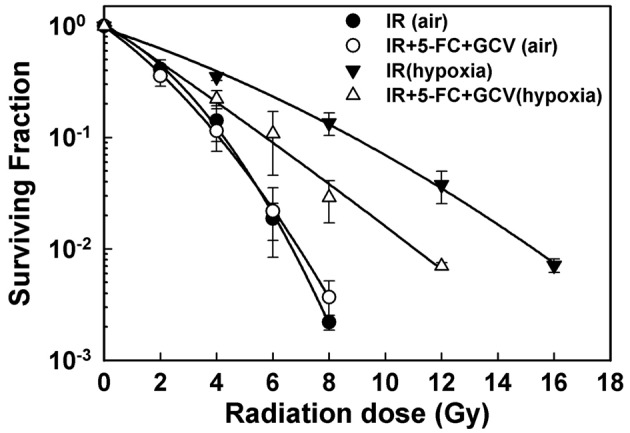
Radiosensitizing effects *in vitro*. HCT8-HRE cells were exposed to 5-FC (0.5 mg/ml) and GCV (5 μg/ml) under normoxic or hypoxic conditions for 48 h, followed by various doses of radiation. Surviving fractions were determined using colony formation assay. Each data point and error bar represents average and SE of three independent experiments. IR, irradiation.

Data on the effect of 48 h GCV and 5-FC treatments on the radiation response of normoxic and hypoxic HCT-HRE cells are presented in Fig. 4. Treatments with GCV plus 5-FC had little radiosensitizing effect in normoxic HCT-HRE cells. However, drug treatments did enhance the radiosensitivity of hypoxic HCT-HRE cells.

### Hypoxia-induced triple suicide gene expression in conjunction with GCV and 5-FC treatments exerts bystander effects

**Figure 5:**
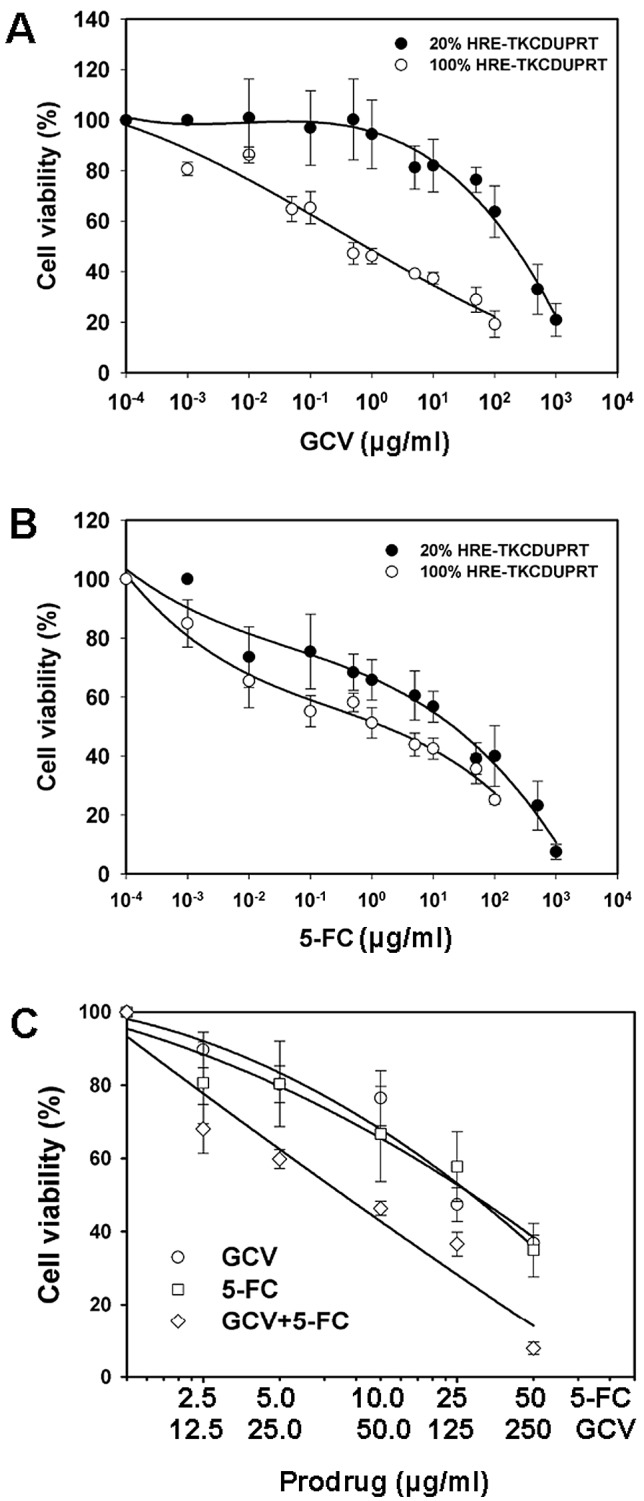
Bystander effects in HCT8-HRE cells. Sensitivity of cell mixture of 20% HCT8-HRE cells and 80% control cells were exposed to (A) GCV or (B) 5-FC alone or (C) in combination of GCV plus 5-FC under 0.5% O_2_ for 48 h. The cell viability was determined by MTT assay.

Bystander killing was examined *in vitro* by culturing mixture with 20% HCT8-HRE cells and 80% control cells and exposing the cells to increasing doses of GCV and/or 5-FC under the hypoxic conditions. As shown in Fig. 5, the cell mixture of 20% HCT8-HRE cells was much less sensitive to GCV than 100% HCT8-HRE cells under the hypoxic conditions (Fig. 5A), whereas 20% HCT8-HRE cell mixtures was slightly less sensitive to 5-FC than 100% HRE cells (Fig. 5B). Furthermore, combined GCV and 5-FC treatments also led to greater cytotoxicity in the mixed cell population (Fig. 5C).

### Hypoxia-induced transgene expression in vivo

**Figure 6:**
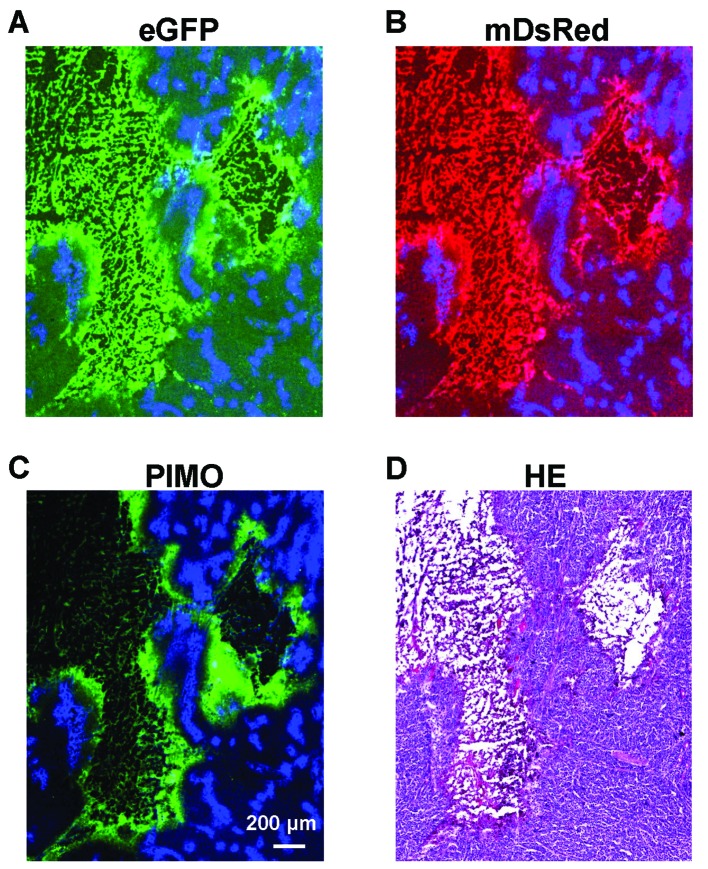
Hypoxia-induced gene expression *in vivo*. Merged images from the same tumor section: (A) eGFP expression (green) and Hoechst 33324 (perfusion, blue); (B) mDsRed expression (red) and Hoechst 33342 (blue); (C) pimonidazole staining (green) and Hoechst 33342 (blue); and (D) HE staining.

Hypoxia-driven transgene expression *in vivo* was validated and compared with distributions of exogenous hypoxia marker (pimonidazole) and blood perfusion marker (Hoechst 33342). Fig. 6 provides a detailed examination and comparison of the different biomarkers in magnified views of a region from an HCT8-HRE tumor. The merged images of eGFP and Hoechst (Fig. 6A) or mDsRed and Hoechst (Fig. 6B) show that eGFP (green) and mDsRed (red) are located between well perfused area (blue) and the necrotic region, consistent with hypoxia-induced expression of the eGFP and mDsRed reporter gene. Fig. 6C shows that the exogenous hypoxic marker pimonidazole (green) is also accumulated in regions of low perfusion.

### Radiation combined with 5-FC and GCV treatments delays tumor growth

**Figure 7:**
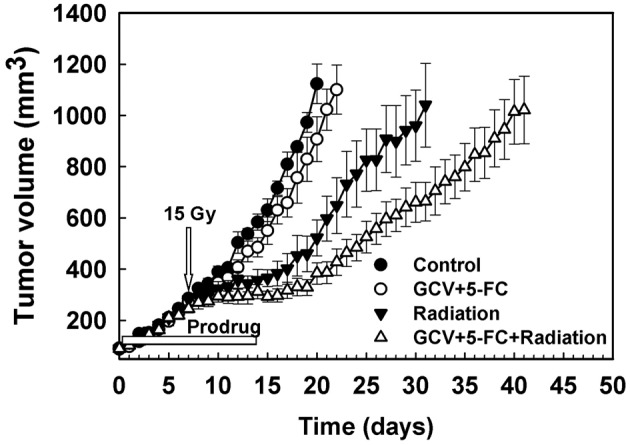
Tumor control effects *in vivo*. Tumor growth kinetics in mice bearing HCT8-HRE tumors are shown as (●) PBS, (○) treated with 14 daily dose of GCV (30 mg/kg) and 5-FC (500 mg/kg), (▼) irradiated 15 Gy on the 5th day, (□) treated with 14 daily dose of GCV (30 mg/kg) and 5-FC (500 mg/kg), followed by 15 Gy of radiation on the 5th day. The error bars are SE from 10 tumors.

The response of the HCT8-HRE tumors to prodrug (GCV and 5-FC) treatment with or without radiation was assessed using the tumor growth delay assay. As shown in Fig. 7, 5-FC (500 mg/kg) and GCV (30 mg/kg) treatments for 14 days alone or radiation (15 Gy) alone, slightly delayed the tumor growth. However, treatments of GCV plus 5-FC combined with irradiation showed significant tumor growth delay (P<0.01).

## Discussion

The hypoxic microenvironment, an important feature of human solid tumors but absent in normal tissue, may provide an opportunity for cancer-specific gene therapy. Studies have demonstrated the potential of hypoxia/HRE-regulated gene therapy for cancer with HSV1-TK (10,18), bacterial CD (7,19,20) and yeast CD (21,22) employed. To explore the possibility of the hypoxia-targeted triple suicide gene therapy strategy, HCT-8 cell lines stably transfected with hypoxia-inducible vectors (HRE-TK/eGFP and HRE-CD/UPRT/mDsRed) were established. Hypoxic induction of the TK/eGFP and CD/UPRT/mDsRed protein (Fig. 1) led to increased sensitivity to GCV and 5-FC (Figs. 2 and 3). At the same time, significant radiosensitizing effects were detected *in vitro* after 5-FC and GCV treatments in the hypoxia conditions (Fig. 4). More importantly, administration of 5-FC and GCV in the tumor-bearing mice in combination with localized irradiation resulted in significant tumor growth delay, as compared with prodrug or radiation treatments alone (Fig. 7). These results clearly demonstrated the potential of hypoxia-targeted radio-gene therapy with the HRE-TKCDUPRT/GCV+5-FC approach.

One potential advantage of suicide gene therapy relevant to clinical application is its radiosensitization effect. Our data clearly showed that the co-expression of hypoxia-regulated HSV1-TK, CD and UPRT triple genes not only significantly increased the sensitivity of GCV and 5-FC, but also improved the radiosensitizing effect of GCV and 5-FC *in vitro* (Fig. 4) and *in vivo* (Fig. 7). It has been shown that HSV-1 TK/GCV gene therapy may inhibit the repair of radiation-induced sublethal DNA damage (23). Others have suggested that the radiosensitization in CD/5-FC and CD/UPRT/5-FC approaches is mediated through the inhibition of thymidylate synthase by 5-FdUMP, resulting in the depletion of deoxythymidine monophosphate pools and increased DNA strand break, as well as redistribution of cells to the radiosensitive early S phase (24). Previously, we demonstrated that the CDUPRT/5-FC approach has greater radiosensitization effect than the CD/5-FC system (13). The hypoxia-regulated CD/5-FC approach has shown significant tumor controlling effects as combined with irradation in different tumor models (19,22). Therefore, it is not surprising that significant tumor control effects were found as the triple suicide gene approach was combined with radiation.

Another advantage of suicide gene therapy approaches is the bystander effects. The HSV1-TK/GCV system requires direct cell-to-cell contacts and active transport of toxic GCV metabolites to neighboring cells through the gap junctions to exert their bystander killing effect (25). On the other hand, in the CD/5-FC system, the converted 5-FU is able to diffuse across the cell membrane into adjacent cells without going through the gap junction, resulting in a more powerful bystander effect (26). Strong bystander effect has also been shown with the CDUPRT/5-FC approach (27). In the present study, the cell mixture of 20% HRE-TKCDUPRT cells was more sensitive to 5-FC than GCV under the hypoxic conditions (Fig. 5), whereas the sensitivity of the 100% HRE cells to GCV and 5-FC was similar as determined by MTT assay (Fig. 3). This is partly due to the low expression of gap junctions in the human colorectal cells (25). Even so, combined GCV and 5-FC treatments leads to greater cytotoxicity in mixed cell populations (Fig. 5C), which demonstrated the superior bystander killing in the hypoxia-driven TKCDUPRT approach.

Several factors may impact on the efficacy of hypoxia-targeted gene therapy. Firstly, the tumors with high hypoxia fraction, for example large tumors, will be targets for hypoxia-regulated suicide gene therapy. In the present study, treatment of 5-FC plus GCV in tumor-bearing mice showed only mild tumor growth delay (Fig. 7). It may be due to low hypoxia fraction in the model used (Fig 6). Secondly, HIF activity in tumors depends on availability of the HIF-1α/2α subunit, the levels of which increase under hypoxic conditions and through activation of oncogenes and/or inactivation of tumor suppressor genes (such as p53). HCT-8 tumor cells bear wild-type p53 gene, which may limit the hypoxia-induced gene expression *in vivo*. To investigate the potential role of hypoxia-targeted triple suicide gene therapy, tumor models with different hypoxia conditions and gene profile were used.

The potential limitation of the present study is the use of stably transfected cell lines and associated tumor models. As two plasmids carrying hypoxia-regulated TK or CDUPRT were used, the hypoxia-induced expression/function and cytotoxicity of TK and CDUPRT were not comparable (Figs. 1 and 2). To translate this strategy to clinical application, we are generating adenovirus vectors to deliver hypoxia-regulated TK, CD and UPRT genes into *in vivo* tumor models and to test whether triple gene transduction following GCV and 5-FC treatment will improve the efficacy of radiotherapy. To overcome the limitations in introducing gene therapy into clinical use, advanced methods of hypoxia-targeted gene delivery are also being developed.

In conclusion, our data suggest that the hypoxia-inducible TK/GCV+CDUPRT/5-FC triple suicide gene therapy may have the ability to specifically target hypoxic cancer cells and improve the tumor control in combination with radiotherapy. This novel model may also serve as a valuable tool to study the correlations among tumor hypoxia, radiation and hypoxia-targeted radio-gene therapy.
